# New sandwich-type polymeric potassium-dicyanoargentate(I) complex: synthesis, characterization and enzymatic activity

**DOI:** 10.3906/kim-2004-42

**Published:** 2020-08-18

**Authors:** Nesrin KORKMAZ

**Affiliations:** 1 Department of Biotechnology, Faculty of Science, Bartın University, Bartın Turkey

**Keywords:** Silver complexes, X-Ray, metabolic enzymes, enzyme inhibition, acetylcholinesterase

## Abstract

Coordination compounds containing dicyanoargentate(I) have remarkable biological potential due to their therapeutic antibacterial, antifungal, antibiofilm, and anticancer properties. In this study, a new dicyanoargentate(I)-based complex was synthesized and characterized by various procedures (elemental, thermal, FT-IR for complex) involving crystal analysis of the complex. In addition, the biological activity of this new compound on the acetylcholinesterase (AChE) enzyme, an important enzyme for the nervous system, was investigated. When the infrared (IR) spectrum of the complex is examined, the OH vibration peak resulting from H_2_O molecules in the structure at 3948-3337 cm^−1^ and at 2138 cm^−1^, along with a CN peak coordinated to Ag, can be seen, indicating that the mass remaining in the thermal degradation of the complex at 1000 ◦ C is the weight corresponding to the metal mixture consisting of K+Ag (calc.: 68.06). The crystal method revealed that the complex has a sandwich-like, polymeric chemical structure with layers formed by K^+^ cations and [Ag(CN)_2_H_2_O]^−^ anions. Therefore, the AChE enzyme has potential therapeutic uses in improving ACh levels in brain cells, in reducing various side effects, and in improving cognitive impairment, especially in advanced Alzheimer’s disease patients. In this study, the activity of this newly synthesized complex on AChE was also investigated. As a result of this research, [Ag(CN)_2_(H_2_O)K] had 0.0282 ± 0.010 μM Ki values against AChE. The compound was therefore a good inhibitor for the AChE enzyme. This type of compound can be used for the development of novel anticholinesterase drugs.

## 1. Introduction

Coordination polymers are compounds obtained by the linking of metal atoms with bridge ligands in an infinite arrangement [1]. The choice of the ligand used in the synthesis of coordination polymers is of great importance because coordination polymers have different structures and properties depending on the ligands they contain. The most important feature of the ligands used for this purpose is the ability to bridge the metal centers. Therefore, these ligand molecules should be multident ligands having two or more donor atoms and which generally contain groups such as N-, O-, S-, or CN-, which have high donor properties. Examples of these bridge ligands are SCN-, CO, N^−^_3_, NCX^−^ (X: O, Se, S), I_2_, NO^−^_3_, and CN^−^ ligands. These ligands are preferred because they can exhibit ambidentate properties and are extensively used in the synthesis of coordination compounds [2,3].

In recent years, a number of cyanido complexes have been characterized by synthesizing a wide variety of transition metals and cyanido ligands. In several comprehensive studies, [M(CN)_4_]^2−^ (M^II^ = Ni, Pd, Pt, Ag, and Au) anions, Ni^II^, Cu^II^, Zn^II^, and Cd^II^ secondary metal ions, as well as a series of bimetallic cyanido containing auxiliary ligands with N and O donor atoms, have been used by researchers to synthesize the complex. The structural characterizations of these complexes, catalysis applications, EPR properties of the d^9^ structure, as well as biological properties, have been determined [4–16].

Silver compounds have been the subject of many studies attracting the attention of scientists because of their many useful properties. When examined in terms of electron distribution, Ag^+^ ion, which is a d^10^ structure, can produce interesting coordination geometry with different supramolecular and molecular structures [17–22]. Since ancient times, experimenting with and using the biological activity of silver has led to more and more experiments and studies. The antibiotic effect of silver compared to conventional antibiotics has been reported to be more effective in various studies [23,24]. Therefore, it may be an alternative for bacteria with high antibiotic resistance [25,26]. Silver not only intitiates activity against bacteria but also affects nanoparticles through antibiofilm action; compounds containing silver that exhibited antitumor activity have also been reported in the literature [27–32]. Furthermore, Ag^I^ compounds have different applications as catalysts in organic synthesis reactions [32,33]. The antibacterial, antibiofilm, antifungal, and anticancer properties of dicyanidosilver(I) complexes have been investigated recently and their potential to be antiagents due to their very good biological activity has been observed [12–14,30–32]. These substances are synthesized quickly, easily, and with high efficiency, so their low cytotoxicity is of great importance for biological studies.

In this study, heteroleptic cyanidoargentate, i.e. the [Ag(CN)_2_(H_2_O)K] complex to which the CN^−^ and H_2_O ligands are linked, has been successfully synthesized (Scheme). This novel heteroleptic complex was characterized by elemental analysis, FT-IR, thermal analysis, and X-ray single crystal analysis. In addition, the inhibition activities of [Ag(CN)_2_(H_2_O)K] complex against acetylcholinesterase (AChE) metabolic enzyme were obtained in this study.

**Scheme:**
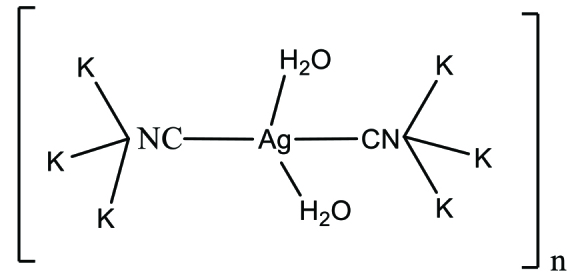
Complex consisting of a reaction of silver nitrate and potassium cyanide.

## 2. Experimental section

### 2.1. Synthesis and characterization

The synthesis was carried out in a water bath at 40 ◦ C for 4 days. The KCN (153 mg, 1.175 mmol) was dissolved in 20 mL of purified water. The previously dissolved water solution of KCN was added to AgNO_3_ solution (200 mg, 1.177 mmol) and dissolved in a mixture of ethanol (10 mL) and water (20 mL). The clear K[Ag(CN)_2_] solution was then stirred for 4 h. The product was filtered and allowed to crystallize in a 40 ◦C water bath. [Ag(CN)_2_(H_2_O)K] crystals were obtained at a high yield 4 days later (yield: 79%). Elemental analysis of the sample was taken according to the standard (C, N, H, and O values) method. The IR spectrum of the sample was taken as powder in the Shimadzu IRAffinity-1 spectrophotometer in the range of 4000–400 cm^−1^. Thermal degradation of the obtained complex was performed with a Hitachi STA 7300 TG/DTG thermal analyzer and analyzed at 5 ◦C/min using a platinum crucible under nitrogen atmosphere at a heating speed in the 25–1000 ◦C temperature range. X-ray single-crystal analysis was performed on a Bruker APEX-II CCD detector. Thecrystal data and test conditions of the complex are presented in Table 1.

**Table 1:** Crystallographic data and structure parameters for the [Ag(CN)_2_(H_2_O)K] complex.

Empirical formula	C_2_H_2_AgKN_2_O
Formula weight	217.03
Temperature [K]	296
Crystal size [mm]	0.11× 0.08 × 0.07
Crystal system	Trigonal
Space group	P3_1_/c
a [Å]	7.3867 (13)
b [Å]	7.3867 (13)
c [Å]	17.598 (4)
α = β [◦]	90.00
γ [◦]	120
V [Å^3^]	831.6 (3)
Z	6
ρcalcd. [g/cm^3^]	2.588
μ [1/mm]	4.26
F(000)	606
θ range [◦]	3.2–26.4
Index ranges	±9, ±9, ±22
Reflections collected	3416
Reflections observed (>2σ)	525
Data/restrains/parameters	574/3/38
R1 (all)	0.052
wR2 CCDC No.	0.185 1958042
Kristolografi doi	10.5517/ccdc.csd.cc23qhn0

The inhibition potency of [Ag(CN)_2_(H_2_O)K] novel complex on AChE activity was measured using Ellman’s procedure (1961) with spectrophotometrically [34]. Acetylthiocholine iodide (AChI) was used as a substrate of the enzymatic reactions and 5,5’ -dithio-bis(2-nitro-benzoic)acid (DTNB) for the estimation of the AChE activities. The reaction mixture consists of 100 mM Tris-HCI (pH = 8), 10 mM AChI, 10 mM DTNB, 15 μL AChE solution, and different concentrations of sample complexes. The absorbance change of the mixture was read at a wavelength of 412 nm.

## 3. Results and discussion

### 3.1. Elemental analysis

Elemental analysis data were found as follows; Anal. Calc. (%) for C_2_HN_2_OAgK: C, 11.12%; H, 0.47%; N, 12.97%; O, 7.41; found: C, 12.08%; H, 0.82%; N, 13.69%; O, 6.96%.

### 3.2. IR spectra

IR spectroscopy is a method used to characterize organic or inorganic compounds. The IR spectrum shows, for example, the fingerprint with absorption peaks corresponding to the frequencies generated by the vibration of the bonds between the atoms forming the substance. Each substance has its own spectrum [35,36]. The most characteristic and distinctive peak in this cyanido complex is the vibration peak of the cyanido group. It is known that free cyanide gives υ (C≡N) vibration peak at 2080 cm^−1^. When the CN^−^ group coordinates to a metal, it gives electrons to the metal with the donor character σ and accepts electrons again by binding (π-receptivity). Since the electron pair introduced to the metal moves away from the σ orbital of the nonbonding (slightly weaker opposing bond character) carbon, the σ-transmittance υ (CN) while the electrons coming back by binding are located in the orbital molecule π^*^, the π-back binding causes υ (CN) to fall. Since the σ-donor character of the CN group is more dominant than the π-acceptor character, it shifts to higher frequencies in coordinated cyanido groups. In addition, depending on the electronegativity of the metal, the oxidation step and the number of coordinations of the vibration frequency υ (C≡N) shifts to a higher area. If both bridges and end cyanido groups are present in the structure, υ (CN) is split in half. The cyanido group, which has a higher wavenumber than the peaks, belongs to the cyanido group, while the cyanido groups that do not participate in the bridge formation are observed at a lower wavenumber [37].

**Figure 1:**
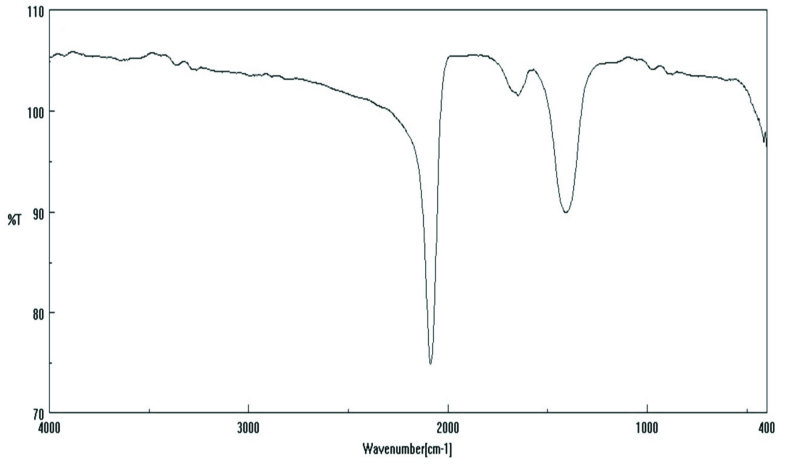
IR spectrum of KCN.

In light of this information, the stretching vibration of the CN peak, which shows chelate, is seen as a sharp peak at 2086 cm^−1^ (Figure 1). The peaks seen in the 1400–1600 cm^−1^ range are thought to belong to the bending vibrations of the CN group.

**Figure 2:**
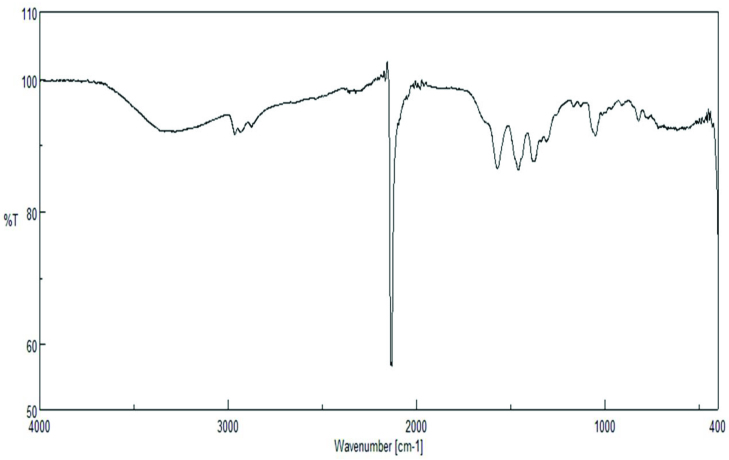
IR spectrum of the complex.

When the IR spectrum of the complex in Figure 2 is examined, the OH vibration peaks at 3288 cm^−1^ and at 2137 cm^−1^ the CN peak coordinated to the metal can be seen. The peaks seen in the 1400–1600 cm^−1^ range are thought to belong to the bending vibrations of the CN group. In addition, the peaks at 600–400 cm^−1^ are thought to be related to the υ (Ag-C), υ (K-N), and υ (Ag-O) vibrations. The presence of this peak in the spectrum and free cyanide observed in our complex shows the formation of shear.

### 3.3. Thermal analyses

**Figure 3:**
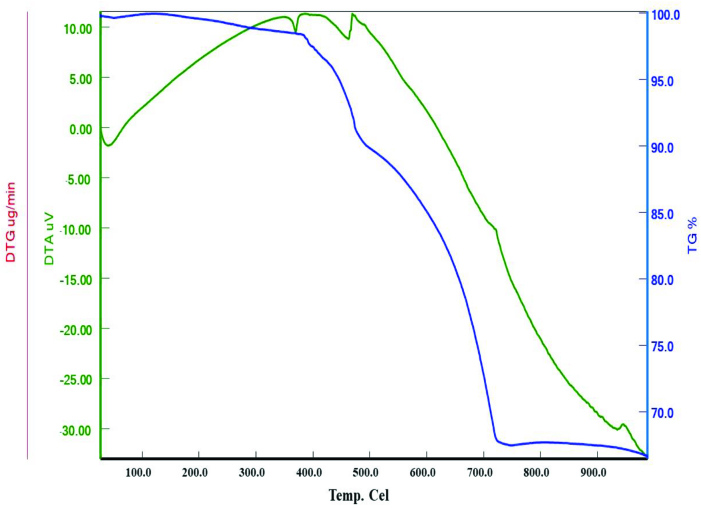
Thermal analyses curves of complex.

Thermal analysis is a method by which certain physical changes in the sample are recorded as a function of temperature or time when heating or cooling according to a predetermined schedule. Thermogravimetric/thermogravimetric derivative–differential thermal analysis (TG/DTG−DTA) measurements of the complex also support the crystal composition as shown in Figure 3.

The TG/DTG curves of the complex are followed by a process in which a two-step weight loss is observed from 30 ◦C to 1000 ◦C. The sharp peak at 160–480 °C in the thermal decomposition graph of complex corresponds to an H_2_O ligand. The H_2_O molecule is attached to the silver atom by a σ bond. Therefore, the bond breaks down, and the decomposition of the water molecule also takes place at higher temperatures. The H_2_O ligand is degraded in the initial steps of the thermal decomposition, which is followed by the thermal degradation of the cyanido ligand. Finally, the final stage of the thermal decomposition is the temperature at which the inorganic components corresponding to the metal residues are located. Experimental data indicate that the mass remaining in the thermal degradation of the complex at 1000 ◦C is the weight corresponding to the metal mixture consisting of K+Ag (calc.: 68.06; found: 67.9).

### 3.4. Crystal structure of complex

**Figure 4:**
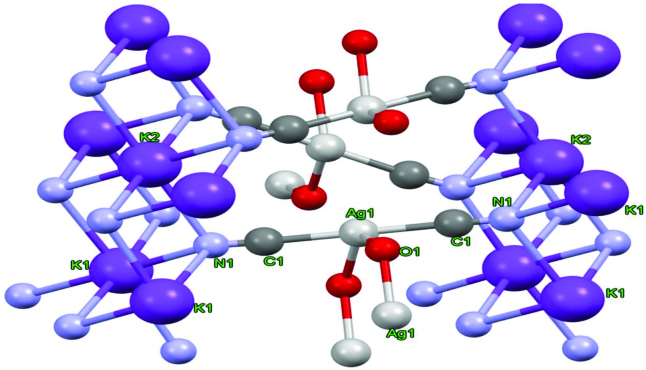
Crystal structure of [Ag(CN)_2_(H_2_O)K] as obtained by low temperature X-ray crystallography.

X-ray crystallography is used to determine the atomic or molecular structure of a crystal. Since salts, metals, minerals, semiconductors, as well as various inorganic, organic and biological molecules can form crystals, X-ray crystallography is important in many scientific fields. The structure of the substance we obtained in our study was illuminated by X-ray crystallography. During single crystal analysis, a heterobimetallic complex (K-Ag) appeared. In addition to the CN ligand in the structure, it formed a heteroleptic dicyanidoargentate complex by binding to Ag(I) as a ligand in the water molecule. The CN ligand acts as a bridge between the Ag and K atoms. Crystal structure analysis reveals that the complex is formed by [Ag(CN)_2_(H_2_O)K] units (Figure 4).

**Figure 5:**
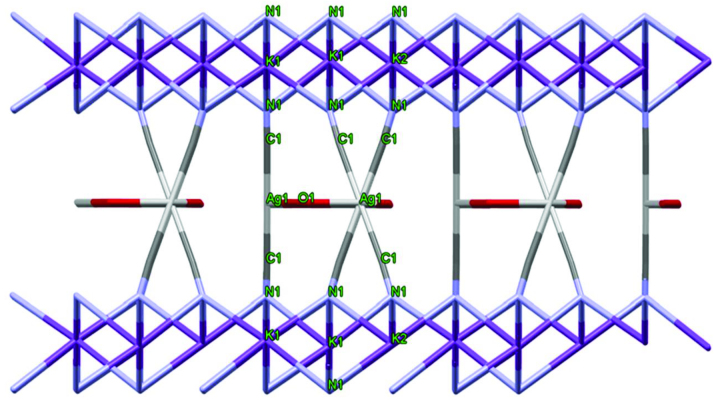
Sandwich-type topology of the complex.

Ag(I) was coordinated with 2 C atoms of bridging cyanido ligands and the O atom of 2 water molecules. Potassium atoms are connected together in the form of cubes intertwined by the binding of N atoms above and below the plane (Figure 5). Here, the K atoms exhibited a structure similar to the 6 coordinated K complexes in the literature [38–40].

**Figure 6:**
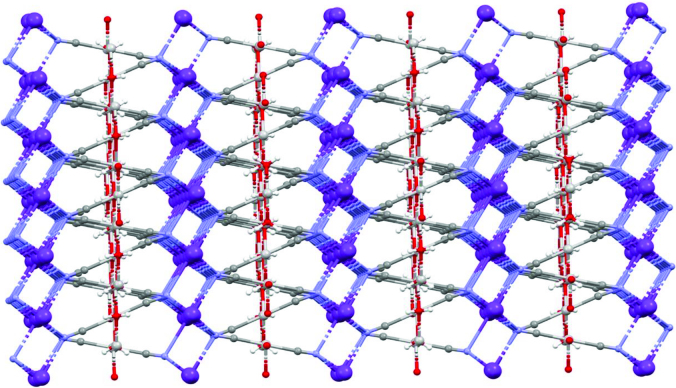
3D configuration view of the complex.

Again, the K atoms above and below the plane are connected to each other by Ag(CN)2 bridges (sandwich-like). Between the K layers, hexagonal intermediate structures consisting of 3Ag + 3O made interesting contributions to the stacking of the complex (Figure 6). The K-N bond distance with 6 coordinated octahedral geometry ranges from about 2.80 to 2.90 Å (Table 2). The angles between N1-K1-N1 and N1-K2-N1 vary between 83.9 (3) and 96.2 (2).

**Table 2:** Selected bond lengths [Å] and angles [◦] for complex.

Bond lengths			
Ag1-C1	2.054(10)	C1-K1^i^	3.468 (11)
Ag1-O1	2.070(11)	K1-N1^iv^	2.823 (9)
K1-N1	2.824(9)	K1-N1^i^	2.906 (9)
K2-N1	2.868(8)	O1-H1	0.84 (2)
Ag1-C1^ii^	2.054(10)	N1-K1^i^	2.906 (9)
Ag1-O1^iii^	2.070(11)	K1-N1^v^	2.823 (9)
Bond angles			
N1-C1-Ag1	175.6(10)	N1^vii^-K1-K2	42.05 (16)
N1-C1-K2	51.8(6)	N1^i^-K1-K2	89.35 (17)
Ag1-C1-K2	125.0(5)	C1^i^-K1-K2	94.4 (2)
N1-C1-K1^i^	51.7(7)	C1^vii^-K1-K2	51.32 (17)
Ag1-C1-K1^i^	126.0(4)	C1^vi^-K1-K2	119.9 (2)
K2-C1-K1^i^	76.5(2)	K2^viii^-K1-K2	119.953 (4)
C1^ii^-Ag1-C1	179.8(6)	N1-K2-N1^vi^	83.8 (2)
C1^ii^-Ag1-O1	110.5(3)	N1^xii^-K2-N1	96.2 (2)
O1-Ag1-O1^iii^	116.2(11)	C1-K2-C1^xi^	71.6 (3)
C1-N1-K1	139.0(9)	N1^iv^-K1-N1^v^	98.4 (2)
C1-N1-K2	110.1(8)	N1^iv^-K1-N1^vi^	83.9 (3)
K1-N1-K1^i^	96.3(2)	N1-K1-C1^i^	98.8 (3)
K2-N1-K1^i^	95.2(3)	N1^v^-K1-C1^vi^	159.1 (3)

Symmetry codes: (i) −x+1, −y+1, −z+1; (ii) −x+y, y, −z+1/2; (iii) −x+y, −x+1, z; (iv) −y+1, x−y, z; (v) −x+y+1, −x+1, z; (vi) x−y+1, x, −z+1; (vii) y, −x+y, −z+1; (viii) x+1, y, z; (ix) x+1, y+1, z; (x) x−y, x, −z+1; (xi) −x+y, −x, z; (xii) −y, x−y, z; (xiii) −x, −y, −z+1; (xiv) −y+1, x−y+1, z.

Although the geometry around Ag(I) is a linear structure formed by the linkage of the bridge CN groups, a geometry near the “T shape” emerges as a result of the binding of H_2_O molecules.

The deviation in T geometry was impaired by the small angle at 69.7(3)◦, which is thought to be caused by steric repulsion and H-bond formation between water molecules (Table 2). The Ag-C bond length of the complex (2.054 Å) is similar to those of dicyanidoargentate(I) complexes observed in the literature [12,30,31].

Intermolecular interaction analysis shows that cyanide N atoms occur between H atoms of the water molecules (Table 3). The formation of the H-bond contributes to better packaging of the structure by providing extra stability to the structure.

**Table 3:** Selected intermolecular distances [Å] and angles [◦] of C3.

D−H···A	d(D–H)	d(H···A)	d(D···A)	D–H···A
O1–H1..· · ·N1^xv^	0.84 (2)	2.60 (3)	3.182 (8)	128 (4)

Symmetry code: (xv) −y+1, −x+1, −z+1/2.

### 3.5. AChE enzyme results

**Figure 7:**
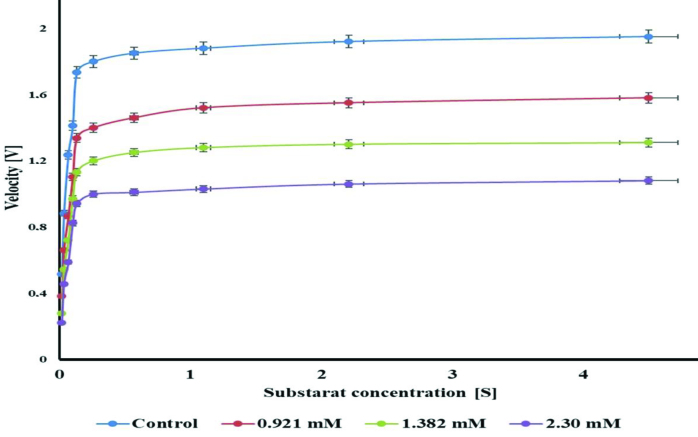
Effect of [Ag(CN)_2_(H_2_O)K] on AChE enzyme inhibition (Michaelis–Menten).

**Figure 8:**
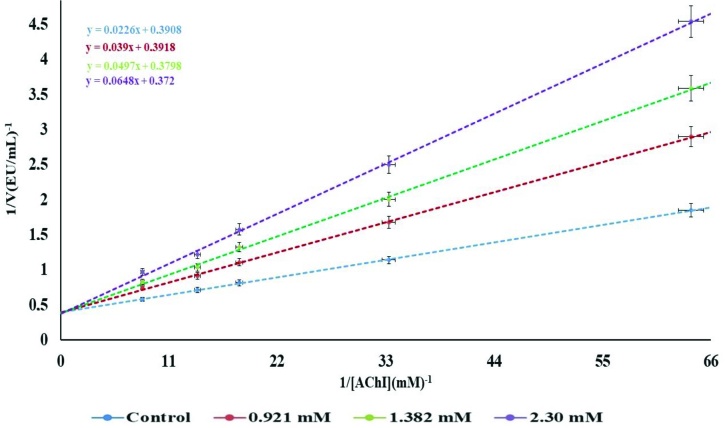
Effect of [Ag(CN)_2_(H_2_O)K] on AChE enzyme inhibition (Lineweaver–Burk).

AChE is found in the brain and in erythrocytes at high concentrations, and it is a crucial enzyme for the nervous system. AChE inhibitors are used in the treatment of several neuromuscular diseases and in the treatment of AD [41]. The half-maximal inhibitory concentration (IC50 ) values of the novel compound demonstrated 50% inhibition of AChE calculated after suitable dilutions. The Ki value of the new compound was determined for AChE. Ki is defined as the binding affinity constant of the novel compound to AChE. To demonstrate the nonallosteric nature of the enzymatic reaction, an enzymatic activity curve (Michaelis–Menten) was first generated. After Vmax stabilization, Lineweaver–Burk graphic studies were performed using inhibitory concentrations of the complex. To determine the Ki values, the novel compound was tested at 3 different concentrations. Michaelis–Menten and Lineweaver–Burk curves were drawn in detail as described previously [41,42]. For descriptions of inhibitory effects, researchers have often used an IC50 value; however, the Ki constant is a more suitable parameter. Ki values were calculated from Lineweaver–Burk graphs (Figures 7 and 8). The novel complex exhibited inhibitory effects on AChE, and the inhibition effect is shown in Figure 6. The Ki constant of AChE was found to be 0.0282 ± 0.010 mM, and IC50 values of the compound against AChE were noted as 1.32 mM (r2 : 0.99). In addition, tacrine (TAC) was used as positive control AChE inhibitor, and it had Ki values corresponding to 392.18 ± 66.28 μM. The IC50 values of these natural compounds and standard (tacrine) showed the following order: TAC (409.10 μM, r^2^: 0.9601) < compound (1.32 mM, (r^2^: 0.99)) for AChE (Table 4). The inhibition type of the complex is competitive against AChE enzyme activity because, while looking at the inhibition mechanism in the presence of the complex, it is possible to say that it shows a competitive inhibition mode from the specific rate graph. Acetylcholinesterase inhibitors (AChEIs) like TAC are commonly used in therapies related to Alzheimer’s disease [43]. Recently, the biological activity of various molecules and metal complexes has been investigated, and research efforts have focused on developing new candidate complexes to investigate their biological potency. As a result, it has been reported that the novel complex worked for the first time, and it has an in vitro inhibitory effect on the AChE enzyme. The inhibitory effect of this molecule on other enzymes should therefore be investigated in different studies.

**Table 4:** Inhibition results of acetylcholinesterase (IC_50_ and Ki values).

Complex	Enzymes	AChE (mM)
	IC_50_	1.32
	r^2^	0.99956
	Ki+std	0.0282 ± 0.010
	Enzymes	AChE (μM)
Standard (tacrine for AChE)	IC_50_	409.10
	r^2^	0.9601
	Ki+std	392.18 ± 66.28

## 4. Conclusion

In summary, the [Ag(CN)_2_(H_2_O)K] complex, consisting of linear CN groups of Ag cations with an electronic configuration of 4d10 , was synthesized and characterized. X-ray spectra exhibited an interesting 3D, sandwichlike structure by hexagonal [Ag(H_2_O)] clusters connecting the infinite [K(CN)_2_] layers of the complex. It was observed from complex TG analysis that it showed a two-step degradation between 30 and 1000 °C. The H_2_O ligand is disrupted in the early stages of thermal decomposition, followed by thermal disruption of the cyanido ligand. After thermal decomposition, a residue corresponding to the Ag + K metal mixture remained in the medium. Coordination polymers containing dicyanoargentate(I) have remarkable biological activities because of their therapeutic properties due to their pharmaceutical properties. Silver-bearing cyanide complex affects the inhibition of the enzyme that plays a role in biochemical reactions that are important for the quality of human life. In this study, we investigated AChE. As a result, the compound showed inhibition at the millimolar levels (1.32 mM, (r2: 0.99)) against the AChE enzyme. The compound was a good inhibitor for the AChE enzyme. This type of compound can therefore be used for the development of novel anticholinesterase drugs.
